# Electronic Spectroscopy of Jet-Cooled NdO

**DOI:** 10.1021/acs.jpca.3c00608

**Published:** 2023-03-17

**Authors:** Joel R. Schmitz, Arianna Rodriguez, Michael C. Heaven

**Affiliations:** Department of Chemistry, Emory University, Atlanta, Georgia 30322, United States

## Abstract

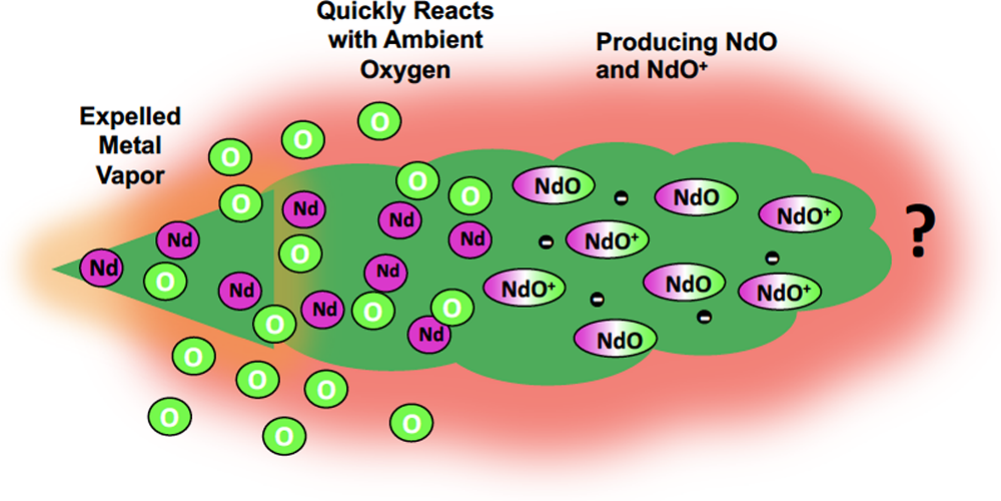

Chemi-ionization reactions of the type M + O → MO^+^ + *e*^–^ (M = Nd or Sm) are currently being investigated as a method to artificially increase the electron density in the ionosphere for control of micro- and radio wave propagation. Experiments involving the release of atomic Nd into the upper atmosphere have resulted in the production of a cloud that, on excitation by solar radiation, emits green light. It has been assumed that NdO was the carrier of this emission, but the existing spectroscopic data needed for this attribution is lacking. While the electronic spectrum of NdO has been well-characterized at wavelengths greater than 590 nm, relatively little spectroscopic data exist for emission wavelengths in the blue-green spectral range. In this study, spectra for jet-cooled NdO were recorded over the range 15,500–21,000 cm^–1^. Rotationally resolved laser induced fluorescence and vibronically resolved dispersed laser-induced fluorescence spectra were recorded, and nine new electronically excited states were identified. The data indicate that the electronic spectrum of NdO has relatively few allowed transitions in the green spectral range, casting doubt on the assignment of the Nd high-altitude release cloud green emission to NdO.

## Introduction

Manipulation of the local electron density in the ionosphere is desirable for control of the propagation of microwave and radiofrequency radiation.^1−5^ The chemi-ionization reactions M + O → MO^+^ + *e^–^* with M = Sm or Nd are being evaluated for their ability to transiently increase the electron density via controlled high-altitude release of the atomic metal vapors.^1−3,6,7^ Sounding rocket experiments have been conducted using both Sm and Nd (referred to as metal oxide space cloud (MOSC) measurements).^1−3,8^ When the rocket reaches the target altitude, the atomic Sm or Nd vapor is released by means of a thermite reaction. The atomic vapor subsequently reacts with the ambient atomic oxygen. The success of this scheme, for the release of electrons, partly relies on the thermodynamics of the chemi-ionization reaction. Initially, the available data indicated that the reactions with both Sm and Nd were exothermic.^9,10^ More recent measurements have shown that the Sm reaction is endothermic by only 0.048 eV.^11−13^ It has also been established that the Nd reaction is 1.76 eV exothermic.^14,15^

The MOSC experiments were conducted under conditions where the cloud was subject to excitation by solar radiation. This resulted in visible-range fluorescence that was described as being pink for Sm and green for Nd.^1,16^ Time-resolved spectra for the Sm release showed blue-region Sm atomic fluorescence immediately after release, with SmO bands developing in the red spectral range at longer times.^2,3^ It appears that there were no spectral features that could be assigned to SmO^+^. Measurements of the electron density indicated a degree of ionization that was far below the expected result.^2,3^

Spectra were not recorded for the Nd release experiments, but it was noted that the green cloud followed the neutral winds. It was surmised that the release had yielded neutral products. This was a surprising result as the thermodynamics predict that the NdO cloud should have a much higher ionization fraction than the SmO cloud. The reaction kinetics also support the expectation that Nd + O will have higher ion yields. Ard *et al.*(6) measured the rate constants for Sm + O and Nd + O over the temperature range 150–450 K. At 300 K, the rate constants were 7 × 10^–12^ and 3 × 10^–10^ cm^3^ s^–1^, respectively.

Analysis of the spectroscopic data from the Sm MOSC experiments has been facilitated by the availability of laboratory data for the electronic transitions of Sm and SmO.^2,3^ Recent work shows that the MOSC red-bands of SmO coincide with strongly allowed, high fluorescent quantum yield transitions observed using laser induced fluorescence (LIF)^17^ and slow electron velocity map imaging (SEVI) techniques.^18,19^ As summarized below, there have been several spectroscopic studies of NdO.^20−25^ However, those investigations primarily focused on bands occurring in the red and far-red spectral ranges. The data for the green spectral range are sparse. For example, Kaledin *et al.*(22) recorded absorption and emission spectra for NdO over the range from 500 to 1100 nm, from which they reported the energies of 69 vibronic band heads. Of these transitions, only two were in the green region (nominally 520–565 nm). Similarly, Effantin *et al.*(21) listed band origins for 45 vibronic transitions, only two of which were marginally in the green region. Hence, without experimental evidence that NdO has suitable transitions, attribution of the green MOSC emission to NdO is questionable. The present study of NdO was undertaken to better characterize bands in the blue-green spectral range, providing a database that can be used for the analyses of future Nd MOSC measurements. In addition, the electronic structure of NdO is a topic of interest in its own right. The density of electronic states is high due to the presence of electrons in the partially filled 4f orbitals, presenting considerable challenges for both experimental and computational investigations (e.g., the lowest energy electronic configuration, Nd^2+^(4f ^3^6s)O^2–^, gives rise to 728 bound electronic states).

Previously, the electronic spectrum of NdO has been characterized using absorption, LIF, thermal emission, and SEVI spectroscopy. Kaledin *et al.*(22) generated gas-phase NdO by heating Nd_2_O_3_ in a furnace to temperatures of 2000–2200 ° C. Absorption and emission spectra were recorded from 500 to 1100 nm. Around 400 bands were observed, and 65 were arranged into transitions originating from 10 different electronically excited upper states. Shenyavskaya *et al.*(25) expanded Kaledin *et al.*’s^22^ characterization of NdO by using near-infrared Fourier transform spectroscopy to obtain rotationally resolved emission spectra from 790 to 1330 nm. Rotational constants for bands previously observed by Kaledin *et al.*(22) were reevaluated, and term energies for new transitions were determined. Four low-lying excited states were observed through transitions with common upper states. Vibrational intervals were determined for the ground electronic state X (1)4 and the (1)3 state. This work established the ground state as an Ω = 4 state, where Ω is the unsigned projection of the electronic angular momentum along the internuclear axis (the electronic state notation is explained below).

Characterization of the low-lying states of NdO was advanced by Linton *et al.*(23) and Effantin *et al.*,^21^ by recording the emission spectra resulting from laser excitation of isolated rotational lines. Transitions to the ground X (1)4 state and nine low-lying states were reported, with four of these states being observed for the first time. Term energies, rotational constants, and, in some cases, vibrational intervals were determined for these states. Finally, magnetic *g*-factors and electric dipole moments for the ground X(1)4 and [16.7]3 state have been determined through Stark and Zeeman splitting measurements.^24^

Babin *et al.*(16) used SEVI anion photoelectron spectroscopy to record vibronically resolved data for NdO. Transitions to vibrational states *v*″ = 0–2 of the X(1)4, (1)5, and (2)4 electronic states of NdO were observed at low detachment energies. States up to 19,681 cm^–1^ (508 nm) above the X(1)4 zero-point level were examined, and 10 correspondences between the SEVI and optical spectroscopy data were identified for the 10,500–16,800 cm^–1^ (952.4–595.2 nm) range.

Computational models for the electronic structure of NdO have been developed using ligand field theory (LFT) and *ab initio* electronic structure models. Carette and Hocquet^26^ used LFT to predict the energies of low-lying states for lanthanide oxides arising from lanthanide 4f ^*N*^, 4f ^*N*–1^5d, 4f ^*N*–1^6s, and 4f ^*N*–1^6p electronic configurations, establishing that the 4f ^*N*–1^6s configuration was responsible for the electronic ground states for the lanthanide oxides, with the exception of EuO and YbO. Kaledin^27^ further developed the LFT model, calculating the term energies and leading electronic configurations of NdO states up to 3 eV. *Ab initio* calculations for the low energy excited states (<1 eV) have been reported by Krauss and Stevens,^28^ Allouche *et al.,*(29) and Babin *et al.*(16)

## Methods

The experimental apparatus used for these experiments has been described previously.^30^ Pulsed laser ablation was used to obtain gas-phase samples of NdO. The beam from a Q-switched Nd:YAG laser was focused onto the surface of a Nd metal rod (American Elements) to generate a pulse of Nd vapor. The 1064 nm fundamental of the Nd:YAG laser was used, typically with a pulse energy near 5 mJ per pulse. A pulsed solenoid valve (Parker Hannifin General Valve Series 9) was used to deliver a synchronous gas pulse to entrain the metal vapor in a carrier gas flow that consisted of 1% N_2_O in He. The gas pulse had a duration of 330 μs and was driven by a source pressure of 120 psi. The mixing of the metal vapor with the carrier flow occurred in a 3 mm diameter channel that had a length of 5 mm. The reactions that formed NdO took place as the gas moved through this channel. At the end of the channel, the reacting gas mixture discharged into a vacuum chamber as a free-jet expansion.

Spectroscopic measurements were carried out using standard pulsed LIF and dispersed laser-induced fluorescence (DLIF) techniques. The dye laser used for these measurements was a Lambda-Physik FL3002e pumped by a Lambda-Physik Compex Pro 201 excimer laser (XeCl, 308 nm). The dye laser had a linewidth of 0.3 cm^–1^ (full width at half maximum) and pulse duration of approximately 10 ns. For a subset of measurements, the laser was operated with the inclusion of an intracavity etalon to reduce the linewidth to 0.06 cm^–1^. The dye laser beam was positioned to cross the free-jet expansion 6 cm from the opening of the reaction channel. LIF, collected using a pair of collimating and refocusing lenes, was viewed along an axis that was perpendicular to both the dye laser beam and the center axis of the free-jet expansion. LIF spectra were detected using a photomultiplier tube (Hamamatsu R955). Long-pass filters were used to reduce the signal from scatted laser light. Simultaneous recording of the spectrum of I_2_ (for wavelengths greater than 500 nm)^31^ or Te_2_ (for wavelengths below 500 nm)^32^ was used to establish absolute wavenumber calibration of the dye laser. A beam splitter was used to send a small fraction of the dye laser beam through a sealed cell that contained either I_2_ or Te_2_ vapor.

A 0.64 m monochromator equipped with a 1200 groves/mm diffraction grating was used for the recording of DLIF spectra. These data were taken by exciting the most intense rotational feature (typically the Q branch) of the vibronic transition of interest, while scanning the monochromator. The sweep was usually over the spectral range defined by energies corresponding to 3500 cm^–1^ lower than the resonant transition to 2000 cm^–1^ higher. In all experiments the signal from the photomultiplier tube was processed by both a boxcar integrator (Stanford Research Systems, SRS 250) and a digital oscilloscope (Tektronix TDS 2014). For lifetime measurements, the fluorescence decay curve was signal averaged for 32 laser pulses and then downloaded directly from the oscilloscope.

### Notation

The electronic ground state of NdO is an Ω = 4 state, specified in the following as X(1)4. The notation (*n*)Ω is used for states with internal energies up to 4000 cm^–1^, where *n* designates the *n*th state for given value of Ω, in ascending energy order. The higher energy excited states of NdO (>10,000 cm^–1^) are labeled using notation previously applied to lanthanide diatomic molecules.^20,21,23,25−27,33^ The upper electronic states are labeled by [*T*_0_/10^3^] Ω, where *T*_0_ is the electronic term energy (including the zero-point corrections) given in cm^–1^ units. Here, we include four digits to obtain adequate specification. Square brackets indicate the energy of the *v’* = 0 level of the upper state. For bands where the vibrational energy of the upper state is uncertain, we use the notation {*T*_*v*0_/10^3^}Ω where *T*_*v*0_ is the energy relative to X(1)4 *v″* = 0 in cm^–1^.

## Results and Analysis

### Visible Range Excitation

**Figure 1:**
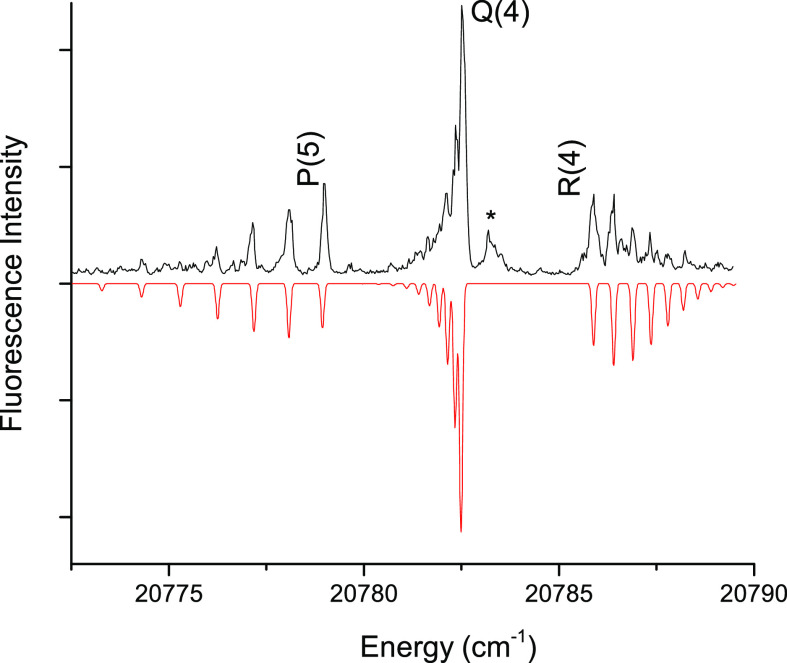
Rotationally resolved LIF spectrum of the NdO {22.44}4-X(1)4 *v*″ = 1 transition. The asterisk (*) marks a feature from a weaker overlapping transition. The downward trace is a simulation generated by PGOHPER.^34^

Rotationally resolved LIF spectra of NdO were obtained in the wavelength ranges of 480–525 and 550–665 nm. Figure 1 shows the rotational structure of a band centered near 20,783 cm^–1^, assigned as the {22.44}4-X(1)4 *v*″ = 1 transition with a rotational temperature of 15 K. Typically, rotational temperatures of 8–20 K were observed for the jet-cooled NdO. It should be noted, however, that the internal energy of NdO was not relaxed to a thermal equilibrium distribution. Vibronic bands that exhibited low rotational temperatures could often be observed coming from lower levels with vibronic energies as high as 2500 cm^–1^. Therefore, both the upper and lower state identities had to be determined for each band. Assignments of the upper and lower state Ω values were established by identifying the first rotational lines in each branch as *J* (the total angular momentum) cannot be less than Ω .^33^ For example, it is evident that the P and R branches in Figure 1 begin with P(5) and R(4). This uniquely defines Ω′ = 4 and Ω″ = 4. Figure 1 includes a simulation of the rotational structure, generated using the PGOPHER software package.^34^ Fitting to the rotational line positions yielded upper and lower state rotational constants (*B′* and *B″*). However, due to the significant cross-correlation of these constants and the small number of rotational lines observed, the absolute errors were quite large (on the order of 0.002 cm^–1^) when both constants were allowed to vary. This compromised the utility of the rotational constants for the purpose of making lower state assignments through comparisons to previously published values.

**Figure 2:**
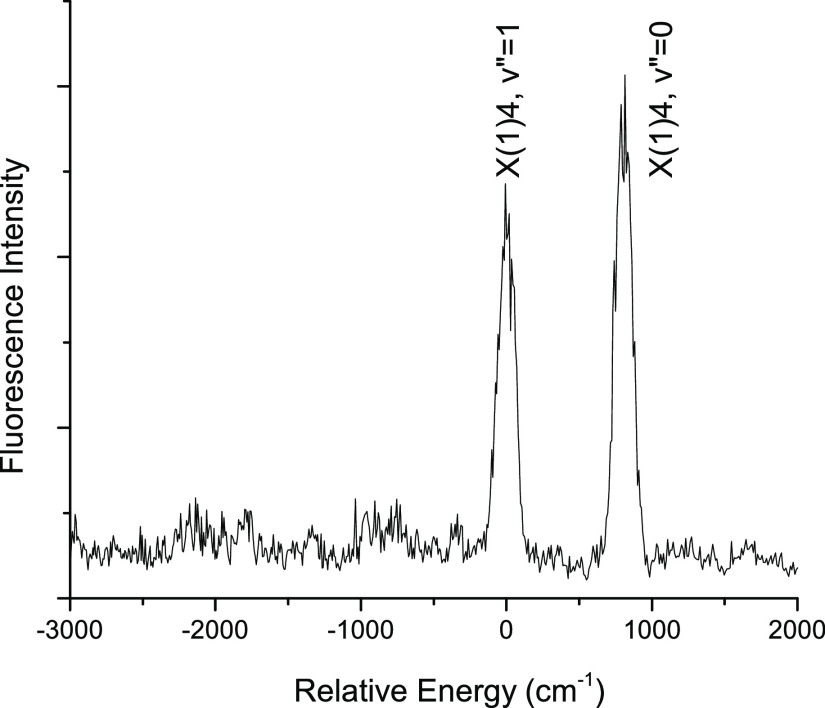
DLIF spectrum recorded using excitation at 20782.9 cm^–1^. The energy scale shows the shift relative to the excitation energy, (υ_emission_ – υ_laser_).

**Figure 3:**
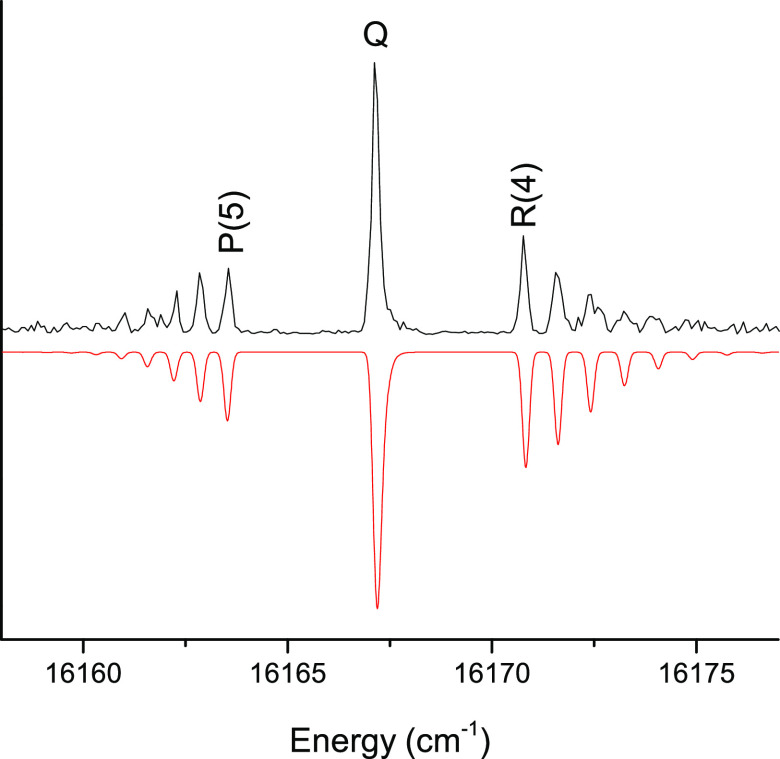
Rotationally resolved LIF spectrum of the NdO {16.17}4-X(1)4 *v*″ = 0 transition. The downward-going trace is a PGOPHER simulation with a rotational temperature of 10 K.

**Figure 4:**
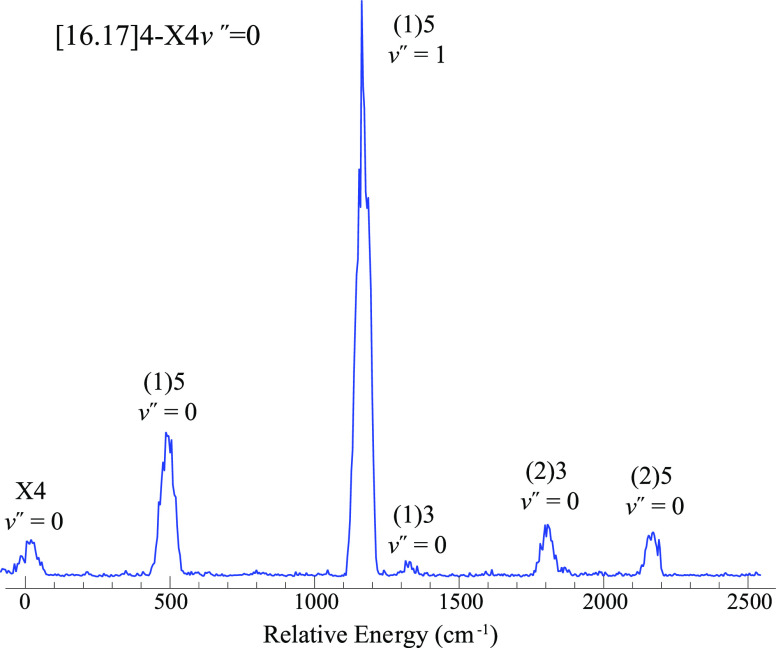
Dispersed fluorescence spectrum induced by excitation of the NdO {16.17}4-X(1)4 *v*″ = 0 transition. Emission features are labeled by their lower state assignments.

Consequently, DLIF spectra were used to identify the lower states using the previously determined pattern of low-lying states. For example, Figure 2 shows the DLIF spectrum recorded using excitation of the band at 20783 cm^–1^ (481.16 nm). The strong emission feature that is shifted approximately 825 cm^–1^ above the excitation band shows that the 20,738 cm^–1^ band must arise from X(1)4, *v*″ = 1, with preferred emission back to the *v*″ = 0 level. Figures 3 and 4 show the LIF and DLIF spectra for a band with an excitation energy near 16167.1 cm^–1^ (618.54 nm). From identification of the first P and R lines, it is established that the transition has Ω′ = Ω″ = 4 The DLIF spectrum exhibits transitions to multiple low-lying electronic states but there were no blue-shifted features, consistent with initial excitation from X(1)4, *v*″ = 0. As expected for the DLIF spectrum from an Ω′ = 4 state, the selection rules restricted the transitions to lower states with Ω″ = 3, 4, or 5.

Once the lower state assignments had been established, the line positions of the rotational bands were fitted to the expression
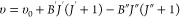
1using PGOPHER.^34^ The lower state rotational constants were held at the literature values for the ^142^NdO isotopologue.^21−23^ The limited range of rotational levels observed did not permit the determination of statistically significant centrifugal distortion constants, and there was no sign of a measurable splitting between the Ω-doublets in any of the bands. The molecular constants resulting from these analyses are collected in Table 1.

**Table 1:** Molecular Constants Obtained from Fitting Rotationally Resolved NdO LIF Bands

transition	*v*_0_ (cm^–1^)^a^	*B′* (cm^–1^)	*B*″ (cm^–1^)	*T*_*v*0_	τ (ns)
[15.63]6-(1)5	15151.30(2)	0.3587(2)	0.3621	15625.0	59
0–0					
[15.63]6-(1)5	15924.23(2)	0.3569(2)	0.3621	16397.9	55(8)
1–0					
{17.22}2-(1)3	16069.65(1)	0.3740(7)	0.3520	17225.7	88(6)
*v*″ = 0					
{16.17}4-X(1)4	16167.03(3)	0.3673(6)	0.3616	16167.0	34(5)
*v*″ = 0					
{18.66}5-(2)4	16245.07(1)	0.3739(1)	0.3496	18658.0	45(5)
*v*″ = 1					
[16.37]3-X(1)4	16737.93(2)	0.3401(1)	0.3616	16737.9	32(5)
0–0					
[18.08]4-(1)5	17537.62(3)	0.3526(5)	0.3606	18841.5	43(5)
1–1					
[18.08]4-(1)5	17601.76(1)	0.35459(9)	0.3621	18075.5	25(5)
0*–*0					
[18.08]4-X(1)4	18075.39(1)	0.3544(2)	0.3616	18075.4	33(5)
0*–*0					
{18.18}3-X(1)4	18175.67(1)	0.3781(2)	0.3616	18175.7	151(22)
*v*″ = 0					
{19.29}5-X(1)4	19287.30(5)	0.3768(7)	0.3616	19287.3	50(5)
*v*″ =0					
{22.04}3-X(1)4	20379.63(3)	0.35264(5)	0.3616	20379.6	46(5)
*v*″ = 0					
{22.44}4-X(1)4	20782.46(3)	0.3367(2)	0.3588	22436.9	78(5)
*v*″ = 2					
{21.61}4-X(1)4	20782.94(1)	0.3387(5)	0.3602	21612.4	26(5)
*v*″ = 1					
{22.01}3-(1)3	20853.16(3)	0.3370(5)	0.352	22009.2	115(5)
*v*″ = 0					

a The uncertainties in parentheses are those obtained from the rotational line fitting. The absolute uncertainties are 0.1 cm^–1^.

**Figure 5:**
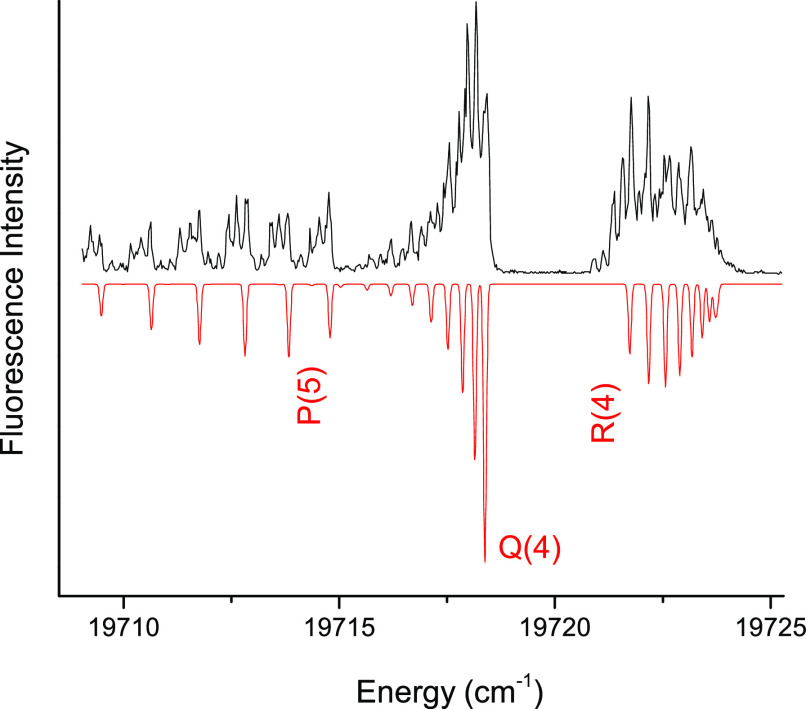
Rotational structure of the {21.37}4-X(1)4 *v*″ = 2 transition. The downward-going trace shows the fit to the ^142^NdO isotopologue.

Although Nd has several naturally occurring isotopes, the most abundant being ^142^Nd (27.2%), ^144^Nd (23.4%), and ^146^Nd (17.2%), isotopic splitting was only resolved for one band. The P-branch lines of the LIF spectrum for this band, presented in Figure 5, are split by the isotopic shifts. For this band, we were able to perform isotopically selective fits for the upper vibronic state, and the resulting molecular constants are collected in Table 2. The isotope shifts were qualitatively consistent with the expected reduced mass dependence, with the bands shifting to lower energy as the reduced mass increased. We could not examine the quantitative agreement with the expected isotope dependence as we do not know the upper state vibrational quantum number.

**Table 2:** Molecular Constants for the {21.37}4-X(1)4, *v*″ =2 Band of NdO

isotopologue	*v*_0_ (cm^–1^)	*B′* (cm^–1^)	*B*″ (cm^–1^)	*T*_0_ (cm^–1^)	τ (ns)
^142^NdO	19718.84(1)	0.3360(3)	0.3588^a^	21373.6(2)	56(5)
^144^NdO	19718.64(1)	0.3356(3)			
^146^NdO	19718.46(2)	0.3342(4)			

a The lower-state rotational constant was fixed at the value given for ^142^NdO by Effantin *et al.*(21) The isotope dependence of the X(1)4 *v*″ = 2 rotational constant was too small to be determined by these measurements.

**Figure 6:**
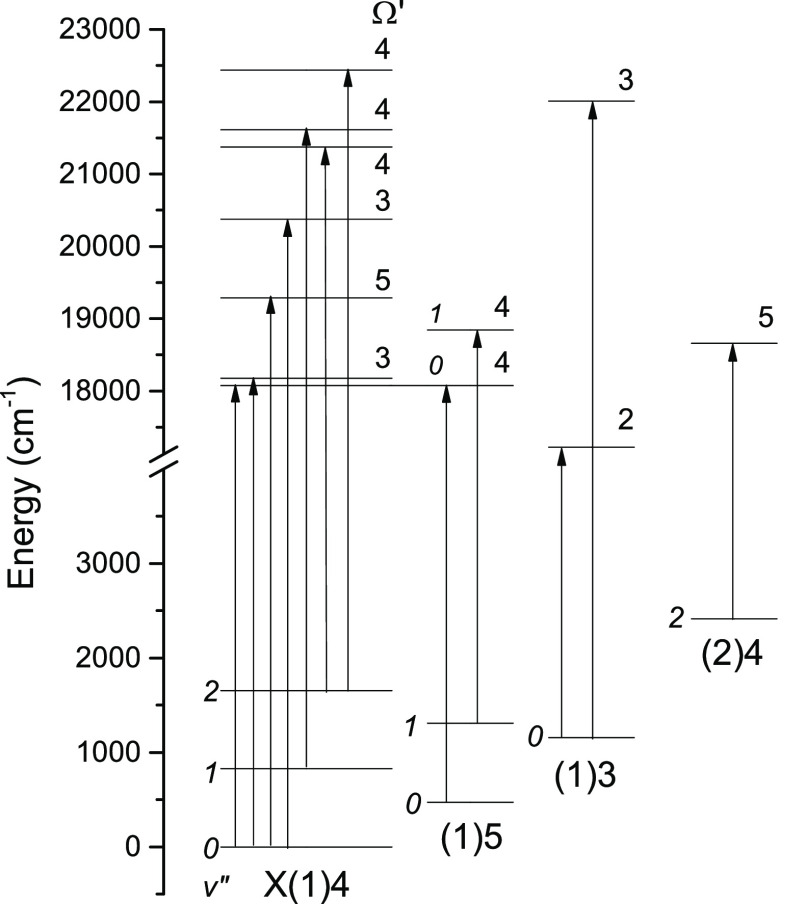
Energy level diagram showing the NdO transitions characterized by LIF in this study. Vibrational quantum numbers, where available, are given in italics.

An energy level diagram showing the upper states characterized in this study is presented in Figure 6. Several bands observed in previous studies^21,22^ were also present in our LIF surveys. Fitting to the rotational line positions of these bands yielded molecular constants that were in good agreement with the published values. However, there was one instance where this was not the case. This was for the band centered near 16,167 cm^–1^ (Figure 3). The origin of this band was listed as being at 16168.376 cm^–1^ by Effantin *et al.*(21) Fitting to our data for jet-cooled NdO yielded a band origin at 16167.1 cm^–1^ (1.3 cm^–1^ discrepancy). The trace in Figure 3 was calibrated against a simultaneously recorded I_2_ spectrum, with an error of, at the most, 0.1 cm^–1^ in the band origin. The data used by Effantin *et al.*(21) was for *J* values above 17, while our data covered the rotational levels *J* = 4–10. Effantin *et al.*(21) stated that the “term energy extrapolated to *J* = 0 is about 16168 cm^–1^.” It seems possible that the extrapolation is responsible for the disagreement.

Many of the DLIF spectra displayed transitions to vibrationally excited levels of the low-lying electronic states. Vibrational progressions were observed for all the states except the (1)2 state, for which just the *v*″ = 0 state was observed. For the (1)5 state, the *v*″ = 0 and 1 states were observed. Due to the low resolution of the DLIF spectra, we could only determine an effective harmonic vibrational constant for each state (the ω_e_x_e_ values were not statistically significant). The results are collected in Table 3.

**Table 3:** Harmonic Vibrational Constants for Low-Lying States of NdO

(*n*)Ω″	ω_*e*_″ (cm^–1^)	*v*″ states used in fit
X(1)4	838(1)	0–5
(1)3	822(8)	0–3^b^
(2)4	825(2)	0–4
(2)3	827(2)	0–5
(2)2	815(2)	0, 2–4
(2)5	831(3)	0–4

As the (1)3 *v*″ = 1 state was not observed in any of our DLIF spectra, the value of 1980.3 cm^–1^ from Linton *et al.*(23) was included to improve the fit.

Lifetimes of the upper states were determined by recording time-resolved fluorescence decay curves. These were all good fits to a single exponential decay, and the resulting lifetimes are listed in Tables 1 and 2.

### Ultraviolet Excitation

In recent work on the spectroscopy of SmO,^17^ we found that the excitation of jet-cooled SmO using 193 nm light from an excimer laser produced an emission spectrum that was remarkably similar to the spectra recorded in the MOSC experiments,^3^ and the chemiluminescent reactions of Sm with oxidants O_3_, N_2_O, NO_2_,^35^ and SO_2_.^36^ Consequently, we examined the emission spectra generated by exciting NdO using 193 and 248 nm light (ArF and KrF lasers). Note that the 193 nm photons (6.4 eV) have sufficient energy to ionize NdO (IE = 5.508 eV^14^) but are not able to photodissociate the molecule (D_0_ = 7.26 eV^15^). The 248 nm photons (5.0 eV) will not ionize or dissociate NdO via one photon absorption.

**Figure 7:**
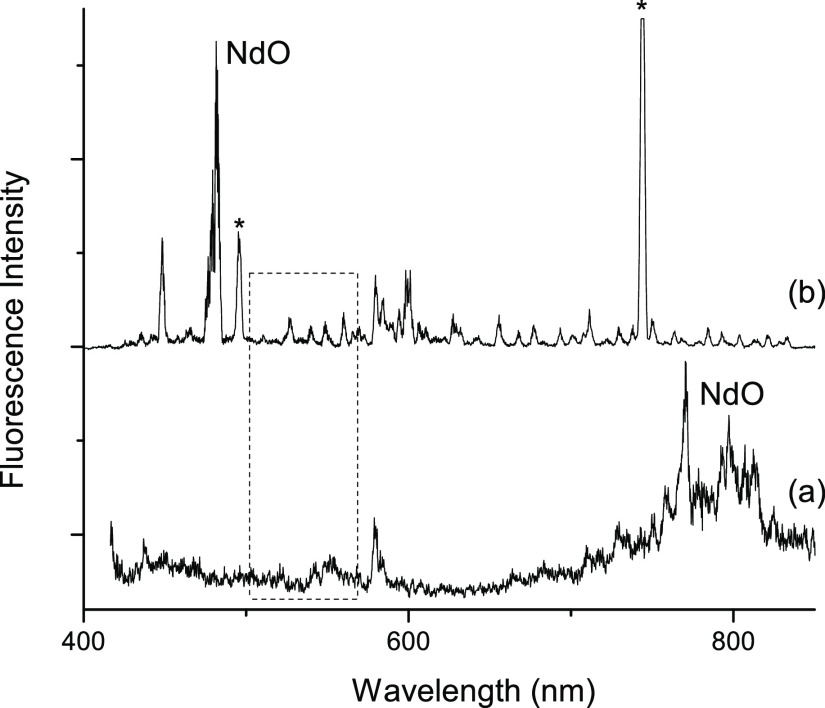
DLIF spectra obtained using UV excitation. Trace (a) shows the result for 193 nm excitation. Trace (b) is for 248 nm excitation. The features marked with asterisks are scattered 248 nm laser light, transmitted in second and third order by the monochromator. The dashed-line rectangle encloses features that emit in the green spectral range.

Excitation of NdO at 193 nm produced weak molecular emissions that were in the and 700–850 nm ranges (Figure 7, trace (a)). The bands in the red spectral range were consistent with the emission bands reported by Kaledin *et al.*(22) It is likely that the emissions from 193 nm excitation ran to wavelengths longer than 850 nm as the response of the PMT dropped rapidly beyond this point.

The spectrum obtained using 248 nm excitation is shown in Figure 7(b). In the visible spectral range, there was only one feature that could be confidently assigned to NdO. This was the most intense band in the spectrum, and it corresponds with the 20,782 and 20,783 cm^–1^ bands seen in the LIF spectra. With regard to the MOSC data, a significant feature of the spectra recorded with both 193 and 248 nm excitation is that they had only weak transitions in the green spectral range (indicated by the dashed rectangle in Figure 7). The carrier(s) of the green emission features were not confirmed to be NdO.

## Discussion

The short radiative lifetimes observed in this study (30–150 ns), combined with the near vertical Franck–Condon factor intensity distributions of the DLIF spectra, indicate that the electronic transitions shown in Figure 6 were strongly allowed. The range of lifetimes was similar to the values for SmO that we found in our recent study.^17^

In total, nine new electronically excited states of NdO have been observed in this study, and a few linkages between these states can be identified. The transitions at 18075.39 and 17601.76 cm^–1^ (553.24 and 568.13 nm) terminate on a common upper state. From the intensity distributions of the associated DLIF spectra, it seems probable that the upper state is a *v*′ = 0 level (and we have adopted this assignment in the labeling of Table 1). The bands at 17601.76 and 17537.62 cm^–1^ then appear to be the 0–0 and 1–1 sequence bands of the [17.08]4–(1)5 transition. Using the lower state energies from ref (21), these assignments yield a Δ*G*_1/2_′ value of 766.1 cm^–1^ for the [17.08]4 state, which is within the typical range for an excited state generated by the promotion of a metal-centered electron (e.g., Nd^2+^(4f ^3^6s)O^2–^ → Nd^2+^(4f ^3^5d)O^2–^).

Kaledin *et al.*(22) organized 65 transitions from their large data set into 10 vibronic band systems. A band with an origin at 15151.33 cm^–1^ (660.01 nm) was assigned as the 0–0 band of system VIII. Subsequently, Effantin *et al.*(21) were able to assign this band as the [15.63]6–(1)5 transition. Kaledin *et al.*(22) also reported a band head for the 1–0 band of system VIII at 15946.7 cm^–1^ (627.09 nm). The system VIII 0–0 band was observed in the present work, and the fitted molecular constants were in good agreement with the literature values. However, the band at 15946.7 cm^–1^ was not present in our data for the jet-cooled molecule. Instead, we found an Ω′ = 6 – Ω″ = 5 band at 15924.23 cm^–1^ with upper and lower state rotational constants that were consistent with assignment to the [15.63]6–(1)5 1–0 transition. With this attribution, the Δ*G*_1/2_′ value for the [15.63]6 state is 772.9 cm^–1^.

In their SEVI study, Babin *et al.*(16) found 10 bands of NdO with internal energies above 10,000 cm^–1^ that could be correlated with transitions observed by means of optical spectroscopy. None of these assigned upper states were more than 16,800 cm^–1^ above the NdO X(1)4 *v*″ =0 level. A further ten bands were observed in the 16,800–19,690 cm^–1^ (595.2–507.8 nm) range. Three of these could be correlated with results from the present study. These were the DD(18,072/18075.4), FF(18,653/18658.4), and KK(19,303/19287.3) bands, where the values in parentheses are the transition energies in cm^–1^ units (ref (16)/present work).

Kaledin^27^ used a LFT model to predict the energies of NdO states in the 0–3 eV range (0–24,200 cm^–1^), providing the only published predictions for states above 10,000 cm^–1^. A striking feature of this calculation is the fact that there are very few states at energies that could be the upper levels for transitions emitting in the green spectral range. The predicted states are mostly clustered below 17,000 (588.2 nm) or above 20,500 cm^–1^ (487.80 nm) (see Tables 1 and 3 of ref (27). We could not find convincing correlations between the states seen above 16,800 cm^–1^ and the LFT predictions. In cases where the energies were similar, there was disagreement for the Ω assignments. However, the LFT model considered only a subset of the possible transitions due to the extreme complexity of the problem.

## Summary and Conclusions

The electronic spectrum of jet-cooled NdO has been examined using LIF and DLIF techniques, with the former achieving rotational resolution. Nine electronically excited states were characterized for the first time. Fluorescence decay measurements yielded radiative lifetimes that were indicative of fully allowed transitions. DLIF spectra, recorded using both visible and UV excitation wavelengths, were used in the process of establishing lower state assignments. These spectra exhibited bands that were primarily in the red and blue spectral regions.

Overall, it is difficult to reconcile the data from the present study with the results from the Nd MOSC field experiments. There is little evidence, from either laboratory spectra or theoretical calculations, to support the assumption that the green emission from the space cloud originated from NdO. Clearly, it should be a priority to record the emission spectra in any future Nd release experiments. In the case of SmO, it was found that laboratory-based studies of chemiluminescent oxidation reactions, such as Sm + N_2_O → SmO* + N_2_, yielded spectra that were remarkably similar to those obtained from the SmO space cloud.^3,35^ Consequently, spectroscopic studies of the chemiluminescent oxidation reactions of Nd may provide an indirect way to probe the carrier of the radiative emission associated with high-altitude Nd release.
